# A Bayesian Mixture Cure Rate Model for Estimating Short-Term and Long-Term Recidivism

**DOI:** 10.3390/e25010056

**Published:** 2022-12-28

**Authors:** Rolando de la Cruz, Claudio Fuentes, Oslando Padilla

**Affiliations:** 1 Faculty of Engineering and Sciences, Universidad Adolfo Ibáñez, Diagonal Las Torres 2640, Building D, Peñalolén, Santiago 7941169, Chile; 2 Data Observatory Foundation, DO, Diagonal Las Torres 2640, Building E, Peñalolén, Santiago 7941169, Chile; 3 Department of Statistics, Oregon State University, 217 Weniger Hall, Corvallis, OR 97331, USA; 4 Departamento de Salud Pública, Facultad de Medicina, Pontificia Universidad Católica de Chile, Santiago 8320000, Chile

**Keywords:** Bayesian inference, MCMC methods, mixture cure rate models, recidivism, Weibull distribution

## Abstract

Mixture cure rate models have been developed to analyze failure time data where a proportion never fails. For such data, standard survival models are usually not appropriate because they do not account for the possibility of non-failure. In this context, mixture cure rate models assume that the studied population is a mixture of susceptible subjects who may experience the event of interest and non-susceptible subjects that will never experience it. More specifically, mixture cure rate models are a class of survival time models in which the probability of an eventual failure is less than one and both the probability of eventual failure and the timing of failure depend (separately) on certain individual characteristics. In this paper, we propose a Bayesian approach to estimate parametric mixture cure rate models with covariates. The probability of eventual failure is estimated using a binary regression model, and the timing of failure is determined using a Weibull distribution. Inference for these models is attained using Markov Chain Monte Carlo methods under the proposed Bayesian framework. Finally, we illustrate the method using data on the return-to-prison time for a sample of prison releases of men convicted of sexual crimes against women in England and Wales and we use mixture cure rate models to investigate the risk factors for long-term and short-term survival of recidivism.

## 1. Introduction

The statistical analysis of survival data is an important topic in several areas, including medicine, epidemiology, biology, demography, economics, engineering and environmental sciences, among others. In these areas, survival analysis has been a well-established method for modeling time-to-event data [1,2,3]. These methods have also been used in the criminological literature since they were first introduced in the field by Ref. [4].

In survival analysis, the survival function S(t) is defined as the probability of observing a survival time *T* greater than some predetermined value *t*; that is, S(t)=P(T>t). It immediately follows that S(t) equals one minus the cumulative distribution function of the survival time *T*, i.e., S(t)=1−F(t). In this context, a standard assumption in survival analysis is that S(t)→0 as t→∞, implying that all observations will eventually experience the event of interest. Unfortunately, this is not a realistic assumption in several applications. For instance, there are many examples in criminal recidivism where a substantial proportion of subjects did not experience the event of interest (return to prison) during their lifetime (after release from prison), and hence, S(t) would plateau to non-zero levels.

To account for the problem of incorrectly assuming that *all* observations will eventually fail, Refs. [5,6,7] developed the so-called split-population models. Similar models, known as *mixture cure rate models*, have been largely discussed in the biometrics literature where the problem is that part of the population is *cured* and will never experience the event of interest, see [8,9,10,11,12,13,14,15,16,17,18,19], among others. These models were popularized in the social sciences by Ref. [20], and alternative formulations and extensions of mixture cure rate models have been proposed more recently by Refs. [21,22,23,24,25,26], among others. For a review of the cure model literature, see [27] and the references therein. At its core, mixture cure rate models include an additional parameter, typically known by biostatisticians as the *cure* factor, which estimates the proportion of the risk set that will never experience a failure (i.e., that will be *cured*). Such an approach to studying recidivism was first introduced to criminology by Ref. [28] and extended by Ref. [6]. Some authors have compared the prediction performance of different statistical models and machine learning techniques using this type of data [29,30,31,32,33,34,35,36,37]. Although there is general disagreement over what is the best modeling approach, traditional statistical models have been shown to perform as well as automatic machine learning models [36].

Criminal recidivism is defined as a relapse to committing a crime or a return to criminal activity. One of the most common measures of recidivism is the percentage of subjects that relapse. With this in mind, recidivism has been studied using different statistical methods, including logistic regression models and survival models. For instance, Ref. [6] considesr a survival analysis approach to criminal recidivism and seeks to determine how some special programs affect the behavior of convicts once they are released. Other approaches to the problem include Ref. [38], where the authors used path analysis to model driving under the influence (DUI) offenses, and Ref. [39], where the authors discussed the advantages of neuronal networks over logistic regression models for prediction. Ref. [40] considered semi-parametric risk models to study recidivism in several states in the USA and argued that each state should be modeled separately. Furthermore, they notice that cure rate models will be successful only if long-term follow-ups are available since medium-term follow-ups (six to seven years) suffer from some estimation problems. In this context, however, Ref. [7] was able to fit such models without any difficulties.

In this paper, we discuss the mathematical details of a general framework for parametric mixture cure rate models and illustrate the usefulness of these models in the study of recidivism data. Specifically, we use the proposed method to analyze data corresponding to a cohort of men convicted of sexual crimes back in 1973 in England and Wales and investigate the covariates affecting their short-term and long-term survival. Further, we compare the performance of the mixture cure rate model with the logistic regression model and Cox regression model, which are also prevalent in recidivism analysis. We fit all the proposed models using a Bayesian framework and estimate all parameters of interest using MCMC methods.

The rest of the article is organized as follows: Section 2 presents the mathematical details of the parametric mixture cure rate models and the use of a logit link and Weibull distribution to model the probability of eventual failure and timing of failure, respectively. In Section 3, we discuss the details of the Bayesian approach and parameter estimation using MCMC methods. In Section 4, we analyze a real dataset to illustrate the advantages and limitations of the mixture cure rate models compared to the standard approaches based on logistic regression and Cox regression models. We end in Section 5 with a brief discussion of the results and directions for future work.

## 2. Mixture Cure Rate Models

Mixture cure rate models are unique in that they do not assume that every subject will eventually experience the event of interest. Instead, these models split the population into two groups—one that will experience the event (susceptible) and one that will not (non-susceptible). For example, in studies of criminal recidivism, some subjects will not return to prison and, therefore, will be part of the non-susceptible group. Typically, non-susceptible subjects are modeled through logistic regression, and their odds of long-term survival are investigated, while the susceptible patients are modeled by a survival model, and their short-term hazard is calculated.

Suppose there are *n* subjects that are followed up over a period [0,L), with 0<L<∞. For each subject i=1,…,n, let $T_{i}>0$ be a random variable representing the survival time that is possibly right-censored. For each *i* define an indicator variable $U_{i}$, taking the values $U_{i}=1$, when the subject *i* is susceptible, and $U_{i}=0$, when the subject *i* is non-susceptible. It follows that $T_{i}$ is well-defined only when $U_{i}=1$.

Given a vector of covariate information $z_{i}={\left(z_{i1},\ldots ,z_{iq}\right)}^{\prime }$, we define $\pi (z_{i})$ as the probability of being susceptible. In other words, $\pi \left(z_{i}\right)=P\left(U_{i}=1|z_{i}\right)=1-P\left(U_{i}=0|z_{i}\right)$. Similarly, we denote by $f(t_{i}|x_{i},z_{i})$, $F(t_{i}|x_{i},z_{i})$ and $S(t_{i}|x_{i},z_{i})$ the corresponding probability density function (pdf), cumulative distribution function (cdf) and survival functions of $T_{i}$ for the entire population, where $x_{i}={\left(x_{i1},\ldots ,x_{ip}\right)}^{\prime }$ is a vector of covariate information that may or may not encompass the same variables as $z_{i}$. Finally, we denote by $f_{s}\left(t_{i}|U_{i}=1,x_{i}\right)$, $F_{s}\left(t_{i}|U_{i}=1,x_{i}\right)$ and $S_{s}\left(t_{i}|U_{i}=1,x_{i}\right)$ the corresponding pdf, cdf and survival functions of susceptible individuals, which may also depend on $x_{i}$.

Assuming that $F_{s}\left(\cdot |\cdot \right)$ is a proper cdf, we have for any $0\leq t_{i}<\infty$

$$P\left(T_{i}\leq t_{i}|U_{i}=1,x_{i}\right)=F_{s}\left(t_{i}|U_{i}=1,x_{i}\right)\;\mathrm{and}\;P\left(T_{i}\leq t_{i}|U_{i}=0,x_{i}\right)=0.$$

On the other hand, the cdf of the entire population is

$$\begin{array}{cc} F(t_{i}|x_{i},z_{i}) & =P(T_{i}\leq t_{i}|x_{i},z_{i})\\ \; & =P\left(T_{i}\leq t_{i}|U_{i}=0,x_{i}\right)P\left(U_{i}=0|z_{i}\right)+P\left(T_{i}\leq t_{i}|U_{i}=1,x_{i}\right)P\left(U_{i}=1|z_{i}\right)\\ \; & =\pi \left(z_{i}\right)F_{s}\left(t_{i}|U_{i}=1,x_{i}\right), \end{array}$$

and the corresponding pdf is $f\left(t_{i}|x_{i},z_{i}\right)=\pi \left(z_{i}\right)f_{s}\left(t_{i}|U_{i}=1,x_{i}\right)$. Note that $F(t_{i}|x_{i},z_{i})$ is an improper cdf with $F(\infty |x_{i},z_{i})<1$. Furthermore, we observe that in terms of the cdf’s F(·|·) and $F_{s}\left(\cdot |\cdot \right)$ the survival functions can be written as $S\left(t_{i}|x_{i},z_{i}\right)=1-F\left(t_{i}|x_{i},z_{i}\right)$ and $S_{s}\left(t_{i}|U_{i}=1,x_{i}\right)=1-F_{s}\left(t_{i}|U_{i}=1,x_{i}\right)$ and, therefore, the marginal (unconditional) survival function of $T_{i}$ for the entire population is given by

(1) $$S\left(t_{i}|x_{i},z_{i}\right)=1-\pi \left(z_{i}\right)F_{s}\left(t_{i}|U_{i}=1,x_{i}\right)=\left(1-\pi \left(z_{i}\right)\right)+\pi \left(z_{i}\right)S_{s}\left(t_{i}|U_{i}=1,x_{i}\right),$$

where $S\left(t_{i}|x_{i},z_{i}\right)\to 1-\pi \left(z_{i}\right)$ as t→∞. Note that the standard survival model (i.e., $\pi (z_{i})=1$ for all $z_{i}$) can be seen as a special case of the mixture cure rate model when there is no non-susceptible proportion.

At this point, it should be noted that different modeling options are available for $\pi (z_{i})$ and $S_{s}\left(t_{i}|U_{i}=1,x_{i}\right)$. For instance, the proportion of susceptible subjects, given by $\pi \left(z_{i}\right)=P\left(U_{i}=1|z_{i}\right)$, can be modeled using a generalized linear model. Possible link functions include the logit, probit and complementary log–log link. Here, we choose the logit link given its interpretability in terms of the odds ratio. That is,

$$\mathrm{logit}\left(\pi \left(z_{i}\right)\right)=\log\left(\frac{\pi (z_{i})}{1-\pi (z_{i})}\right)=\beta_{0}+\beta_{1}z_{i1}+\ldots ,\beta_{p}z_{ip}=z_{i}^{\prime }\beta ,$$

where β is the vector of regression parameters associated with $z_{i}$. For the survival function, $S_{s}\left(t_{i}|U_{i}=1\right)$, a number of parametric/semi-parametric models have been proposed. Among the parametric models, exponential (Exp), Weibull (WB), lognormal (LN), loglogistic (LL) and Gompertz (GP) are commonly used to model survival data. Their survival functions can be written as

(2) $$S_{s}\left(t_{i}|U_{i}=1,x_{i}\right)=\left\{\begin{array}{c} \begin{array}{cc} \exp\left\{-\exp(\log t_{i}-\mu )\right\}, & \left(\mathrm{Exp}\right)\\ \exp\left\{-\mu t_{i}^{r}\right\},r=1/\sigma >0 & \left(\mathrm{WB}\right)\\ 1-\Phi \left(\frac{\log t_{i}-\mu }{\sigma }\right),\sigma >0\; & \left(\mathrm{LN}\right)\\ {\left\{1+\exp\left(\frac{\log t_{i}-\mu }{\sigma }\right)\right\}}^{-1},\sigma >0 & \left(\mathrm{LL}\right)\\ \exp\left\{\frac{\sigma }{\mu }\left(1-e^{\mu^{2}}\right)\right\},\mu >0,\;\;\sigma >0 & \left(\mathrm{GP}\right) \end{array} \end{array}\right.$$

where μ and σ are the location and scale parameters in the parameterization for the LN, LL and WB distributions. Observe that when r=1, the Weibull distribution reduces to the exponential distribution. In order to add covariates to these parameterizations, in (2) we replace μ with $\mu_{i}=\exp\left(x_{i}^{\prime }\alpha \right)$, where α represents the vector of unknown regression parameters. Among the many possible families of distributions that can be used, the Weibull model offers good flexibility in that it enables a monotonic increasing or decreasing recidivism rate for the susceptible group.

### Likelihood Function for the Mixture Cure Rate Model

Data are denoted as $d_{n}=\left\{\left(t_{i},c_{i},x_{i},z_{i}\right),\;\;i=1,\ldots ,n\right\}$, where $t_{i}$ is the observed survival time for subject *i*, $c_{i}$ is the censoring indicator equal to 1 if $t_{i}$ is uncensored (i.e., observed) and $x_{i}$ and $z_{i}$ are the two covariate vectors defined earlier. We have that for subject *i*, the contribution to the likelihood is $\pi \left(z_{i}\right)f_{s}\left(t_{i}|U_{i}=1,x_{i}\right)$, when $c_{i}=1$, and $\left(1-\pi \left(z_{i}\right)\right)+\pi \left(z_{i}\right)S_{s}\left(t_{i}|U_{i}=0,x_{i}\right)$, when $c_{i}=0$. It follows that the observed likelihood function $L_{obs}$ can be written as

(3) $$L_{obs}\left(\theta |d_{n}\right)=\prod_{i=1}^{n}{\left[\pi \left(z_{i}\right)f_{s}\left(t_{i}|U_{i}=1,x_{i}\right)\right]}^{c_{i}}{\left[\left(1-\pi \left(z_{i}\right)\right)+\pi \left(z_{i}\right)S_{s}\left(t_{i}|U_{i}=0,x_{i}\right)\right]}^{1-c_{i}}.$$

Again, if there is no non-susceptible proportion, $\pi (z_{i})=1$ and the mixture cure rate likelihood function matches the likelihood of the standard survival model.

On the other hand, since $U_{i}$ is the random variable denoting the susceptible ($U_{i}=1$) or non-susceptible ($U_{i}=0$) groups, it follows that when $c_{i}=1$, $u_{i}=1$, and when $c_{i}=0$, $u_{i}$ is unobserved (i.e., it is either 0 or 1). Then, given $U=(u_{1},\ldots ,u_{n})$ the complete likelihood function $L_{c}$ is given by

$$L_{c}\left(\beta ,\alpha ,r|d_{n},U\right)=\prod_{i=1}^{n}\pi {\left(z_{i}\right)}^{u_{i}}{\left(1-\pi \left(z_{i}\right)\right)}^{\left(1-u_{i}\right)\left(1-c_{i}\right)}f_{s}{\left(t_{i}|U_{i}=1,x_{i}\right)}^{u_{i}c_{i}}S_{s}{\left(t_{i}|U_{i}=1,x_{i}\right)}^{u_{i}\left(1-c_{i}\right)},$$

where $f_{s}\left(t_{i}|U_{i}=1,x_{i}\right)$ and $S_{s}\left(t_{i}|U_{i}=1,x_{i}\right)$ denote the pdf and survival function, in this case, corresponding to the Weibull distribution.

## 3. Bayesian Analysis

In order to use a Bayesian framework to fit the mixture cure rate model described in Section 2, we need to specify prior distributions for the parameters in the model. Here, we consider the parameters β, α and σ of the Weibull model and assume that

$$\beta ∼N_{p}\left(b,B\right);\;\alpha ∼N_{q}\left(a,A\right);\;\mathrm{and}\;\sigma =1/r∼IG\left(s_{1},s_{2}\right),$$

where $N_{p}\left(\cdot ,\cdot \right)$ and $N_{q}\left(\cdot ,\cdot \right)$ denote the multivariate normal distributions of dimensions *p* and *q*, respectively, and $IG(s_{1},s_{2})$ denotes the inverse gamma distribution with shape parameter $s_{2}$ and scale parameter $s_{2}$. We further assume that α,β and σ are mutually independent. The hyperparameters $\left(b,B,a,A,s_{1},s_{2}\right)$ can be chosen to be known based on prior information about the problem or taken in such a way that we get non-informative priors when no (or minimal) prior information is available.

Unfortunately, closed-form expressions for the posterior distributions are not available for this model, so we consider sampling-based approximations obtained via Markov Chain Monte Carlo (MCMC) methods. Specifically, the model can be fit by adapting a Gibbs sampler algorithm for mixture models [41,42]. In practice, one shall sample the latent indicators from their posteriors and then use the complete likelihood conditionally on the latent indicators. The general iteration of the algorithm we propose for the model described previously is:

- Sample $U_{i}$, i=1,…,n, from
  $$P\left(U_{i}=0|c_{i}=0\right)=\frac{\pi (z_{i})}{1-\pi \left(z_{i}\right)+\pi \left(z_{i}\right)S_{s}\left(t_{i}|U_{i}=1,x_{i}\right)}$$
- Sample β from
  $$\pi \left(\beta |U\right)\propto L_{c}\left(\beta ,\alpha ,r|d_{n},U\right)\cdot \pi \left(\beta \right)\propto \prod_{i=1}^{n}{\left(\frac{e^{z_{i}^{\prime }\beta }}{1+e^{z_{i}^{\prime }\beta }}\right)}^{u_{i}}{\left(1-\frac{e^{z_{i}^{\prime }\beta }}{1+e^{z_{i}^{\prime }\beta }}\right)}^{\left(1-u_{i}\right)\left(1-c_{i}\right)}\cdot \pi \left(\beta \right)$$
- Sample α from
  $$\pi \left(\alpha |\beta ,U\right)\propto L_{c}\left(\beta ,\alpha ,r|d_{n},U\right)\cdot \pi \left(\alpha \right)\propto \prod_{i=1}^{n}{\left(e^{x_{i}^{\prime }\alpha }\right)}^{u_{i}c_{i}}{\left(e^{-e^{x_{i}^{\prime }\alpha }t^{r}}\right)}^{u_{i}}\cdot \pi \left(\alpha \right)$$
- Sample *r* from
  $$\pi \left(r|\alpha ,\beta ,U\right)\propto L_{c}\left(\beta ,\alpha ,r|d_{n},U\right)\cdot \pi \left(r\right)\propto \prod_{i=1}^{n}{\left(rt^{r}\right)}^{u_{i}c_{i}}{\left(e^{-e^{x_{i}^{\prime }\alpha }t^{r}}\right)}^{u_{i}}\cdot \pi \left(r\right).$$

Note that the MCMC analysis is performed by sampling from the conditional distributions of the parameters. Unfortunately, the complexity of the mixture cure rate models is such that the conditional distributions of the parameters of interest do not have an explicit form but can be sampled from MCMC algorithms such as Metropolis Hastings within the Gibbs sampler. A simple computational approach to simulate these samples can be implemented using the JAGS software (https://mcmc-jags.sourceforge.io, accessed on 17 June 2022), where we only need to specify the likelihood and the prior distributions of the parameters. Although the likelihood function for this specific model is not directly implemented in JAGS, the “zeros trick” approach (based on a Poisson distribution) can be used to specify it indirectly, as discussed in Ref. [43].

For model comparisons, we use the deviance information criterion (DIC), which combines a measure of model fit and a measure of model complexity [44]. Specifically, if Θ denotes the vector of all unknown model parameters, then the DIC is defined as

$$\mathrm{DIC}=2\bar{D}+D\left({\bar{\Theta }}_{M}\right)$$

where the vector ${\bar{\Theta }}_{M}$ contains the mean values of all parameters in the model (M). Therefore,

$$\begin{array}{cc} D({\bar{\Theta }}_{M})= & -2\log f(y|{\bar{\Theta }}_{M},M),\\ \bar{D}= & -2\int \left[\log f\left(y|\Theta_{M}\right)\right]f\left(\Theta_{M}|y,M\right)\;d\;\Theta_{M}\\ = & \;\;\;E_{\Theta_{M}|y}\left(D(\Theta_{M})\right) \end{array}$$

where $\Theta_{M}$ is the sample values of all unknown parameters in model M in a given MCMC iteration. As a result, lower values of the criterion indicate better-fitting models. It should be noted that the DIC is approximately equivalent to Akaike’s information criteria in models with negligible prior information [44]. Further details on the use of the DIC for model comparison are given in [45].

## 4. A Real Data Example

To illustrate the method, we use data from [46], who looked at sexual crimes against women back in 1973 in England and Wales. The sample has 3068 individuals and their corresponding records since 1963. All the cases in the sample were followed-up until 1994. For the analysis, recidivism means that a new conviction occurred. We considered two variables to summarize criminal history: the number of prior convictions for non-sexual crimes in the previous ten years (NP); and the number of prior convictions for sexual crimes in the same period of time (NPS). An indicator variable (UND16) was used to indicate whether the 1973 conviction was due to a crime committed against a victim 16 years old and older (coded as 0) or if the victim was under the age of 16 (coded 1). Lastly, we considered as a fourth variable the age of the individual.

We fit three different versions of the Weibull mixture cure rate model introduced in Section 2: *Weibull SPM${}_{1}$*, in which we do not include covariate information at the split-levels; *Weibull SPM${}_{2}$*, in which we include covariate information at the level $\pi (z_{i})$; and *Weibull SPM${}_{3}$*, in which we include covariate information at both $\pi (z_{i})$ and $S_{s}\left(t_{i}|U_{i}=1,x_{i}\right)$ levels. In addition, we considered the standard Weibull model which does not account for non-susceptibles.

For all the cases, we considered a burn-in-sample of size 10,000 to wash out the effect of the initial values. After the burn-in-sample period, we generated 500,000 samples (using the Gibbs sampling algorithm described earlier) and kept every 100th sample, producing a final sample of 5000 simulated values used to estimate posterior distributions and parameters of interest. Convergence was monitored using trace plots of the generated samples.

**Figure 1:**
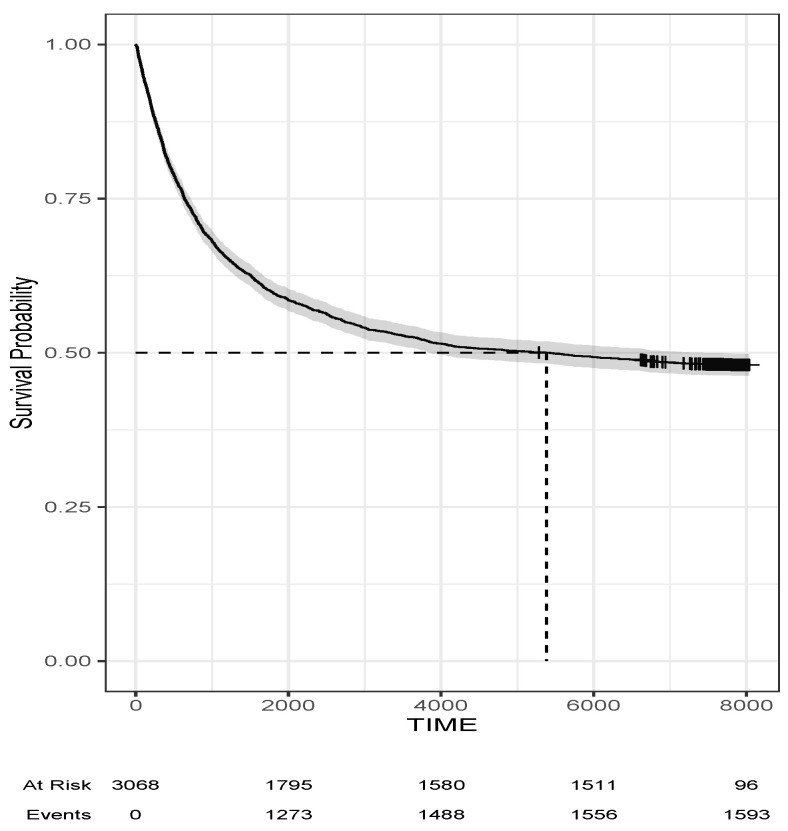
Overall Kaplan–Meier survival curve for the men convicted in the recidivism data example.

The average age of the subjects in the study was 29.5 years. Among them, 14.6% were under the age of 16, 35.4% were between 16–25 and 28.2% were over 35. In the sample, 59.1% of the subjects had no prior convictions and only 5% had more than 5 prior convictions. About 29.7% had committed a sexual crime, and out of them, 51.9% were subsequently repeat offenders. From them, 27.2% committed the same type of sexual offense. Figure 1 displays the overall Kaplan–Meier survival curve for the men convicted. The 95% credible intervals are shown in gray. Note that at the beginning of the study, we had 3068 men at risk of recidivism, while towards the end, we only have 96 subjects at risk of recidivism and 1593 cases of recidivism. The plateau points out the presence of the cure fraction of the men, where about 48% of men did not recidivate during the period of study.

**Figure 2:**
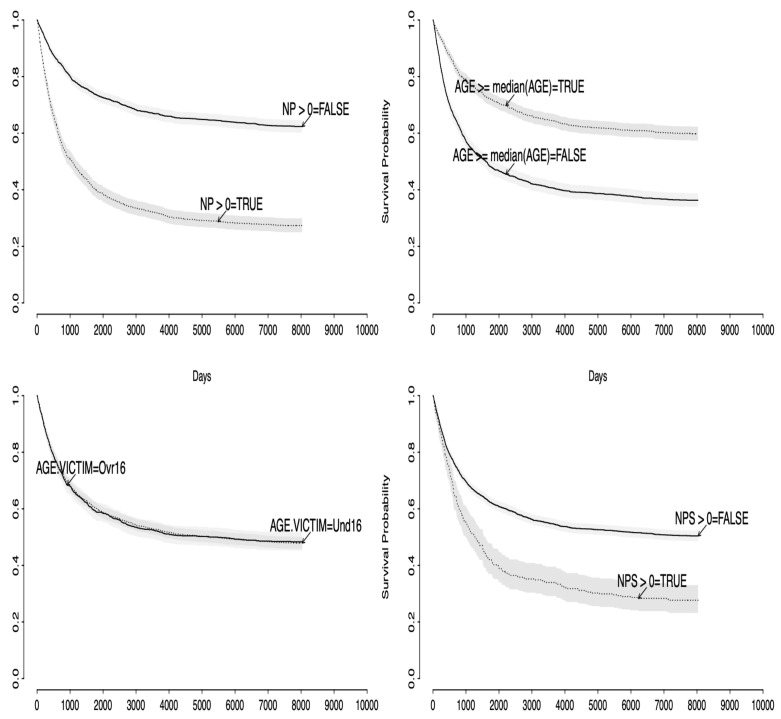
Survival function estimates using the Kaplan–Meier estimator considered in the recidivism data example.

In Figure 2, we find the Kaplan–Meier survival function estimates with the corresponding 95% credible intervals in gray for four different cases determined by the covariates NP, NPS, UND16 and AGE. We considered the following groups: NP (NP > 0 vs. NP = 0), NPS (NPS > 0 vs. NPS = 0), UND16 (Age Victim ≥ 16 vs. Age Victim < 16) and AGE (AGE ≥ median(AGE) vs. AGE < median(AGE)), where the median of the age of the individual is 24.58 years old. Notice that the plot suggests that all covariates NP, NPS and AGE can be useful to estimate the risk of recidivism but that UND16 may not play a significant role in the analysis.

Table 1 summarizes the results of the four models described earlier. Overall, the Weibull SPM${}_{3}$ model, which considers covariates in the survival and logit function levels, does a better job fitting the data based on the DIC criterion. Interestingly, we also note that the mixture cure rate model (fitted with and without covariates) does a better job fitting the data than the standard Weibull model, which does not account for non-susceptibles.

The estimated probabilities of recidivism and survival curve estimates for the Weibull mixture cure rate model are shown in Table 2. We note that the credible interval for the coefficient for the variable NPS (number of prior sexual crimes) contains zero, suggesting that this variable does not contribute to explaining the probability of recidivism. The other variables show the same relationship observed for the probability of recidivism. The number of prior non-sexual crimes increases the probability of recidivism, and having committed the crime against a minor under the age of 16 increases the probability of recidivism. On the other hand, the probability of recidivism decreases as the subject gets older.

The survival component of the mixture cure rate model only applies to those who repeat offenses, because of which neither the number of prior sexual offenses nor whether the victim was 16 and over or under 16 years of age in 1973 influences the risk of recidivism, given that both credibility intervals contain zero. In contrast, the risk of recidivism increases with the number of prior non-sexual crimes and decreases as age increases.

In addition, the results of the fitted model yield an estimate of *r* equal to 0.87, with a 95% credible interval $\left(0.83,0.9\right)$, indicating that the time distribution is not exponential and, therefore, risk is decreasing instead of constant.

Table 3 shows the estimate odds ratio (OR) and hazard ratio (HR). The odds of long-term survival for a victim under the age of 16 were higher than those of 16 years old and older, with estimated values OR =1.38 and a 95% credible interval (1.15,1.65). The OR =1.70 and CI (1.58, 1.84) for NP indicates that for a one-unit increase in the number of prior convictions, the increase in the odds of getting into a recidivism class is 70% while being older decreases the odds of recidivate with OR =0.67 and CI (0.63, 0.71). On the other hand, the hazard of recidivating when the victim is under the age of 16 is higher than those of 16 years old and older, with HR =1.07 and CI (0.31,3.85). Men convicted with prior convictions increase the hazard of recidivate with HR =3.00 and CI (2.38,3.74), while being older decreases the hazard of recidivate with HR =0.20 and CI (0.11,0.36).

To compare our results to other standard analyses, we also fit logistic and Cox regression models to the data. For the logistic regression, we considered the response variable Y=1 if the individual recidivates in a period shorter than a given time period (zero otherwise). The study considers three different time periods yielding three different models: the entire length of the study (corresponding to 21 years of follow-up), at 10 years and at 3 years.

The logistic regression model for final recidivism (at 21 years) gives that the number of prior sexual crimes is not related to long-term recidivism (β=0.14 with a 95% credible interval containing zero). In contrast, the other three variables show evidence of association with recidivism (the corresponding 95% credible intervals do not contain zero). Further, we observe that the greater the number of prior non-sexual criminal convictions, the higher the probability that the individual recidivates (β=0.53). If the crime in 1973 was against a person under 16 years of age, the probability of recidivating was higher (β=0.31), and the probability of recidivating tends to decrease with age (β=−0.04). Recidivism at 10 and 3 years follow a similar pattern.

**Figure 3:**
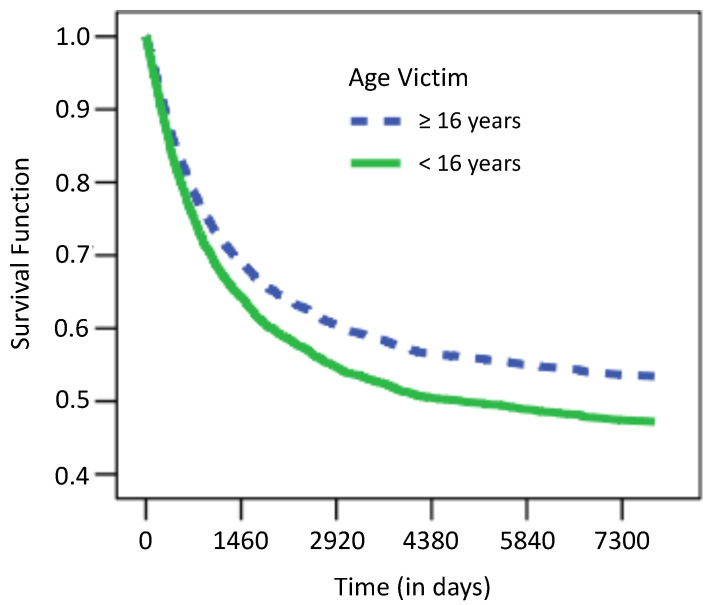
Dependence of survival function of the age of the victim for the recidivism example. Note that the time axis is divided into four-year periods.

For the Cox regression model, only four variables were significant: if the crimes committed by the individuals in 1973 were against persons under 16 years of age, the individuals tend to recidivate more rapidly. Similarly, higher rates of recidivism are observed among those that committed crimes against persons under the age of 16, as shown in Figure 3.

Table 4 presents the general probabilities of recidivism (at the end of the study), as well as at three and ten years for the three models considered in the study.

We observed that 51.9% of the subjects had recidivated by the end of the follow-up. The logistic regression produces a closer estimate (52%) compared to the Cox model, which underestimated this value by over 4%. Note that the mixture cure rate model overestimated this quantity by 0.7%. For the 10-year time period (recidivism at 47.5%), both the logistic regression and Cox models gave similar estimates, improving over the mixture cure rate model that overestimated the rate by 0.6%. At three years, the estimations of the logistic regression and Cox regression models were identical, but the mixture cure rate model overestimated recidivism by 1.7%.

The real prevalence of recidivism at ten years was 33.1% among those who did not have prior convictions for sexual crimes. In this case, all three models over-estimated this value, with the logistic regression showing the best performance (2.8% difference), followed by the mixture cure rate model (3.7% difference). In this case, the Cox model had the worst performance, with a difference of 6.9%.

## 5. Discussion

The data we considered in this in this article were originally discussed in [46]. These data are particularly appealing for the analysis because follow-up times are among the longest available in the recidivism literature allowing proper evaluation of the proposed models in three different scenarios: the long-term (21 years), medium-term (10 years) and short-term (3 years). The results obtained in our analysis suggest that there is no substantial dominance of any model over the others in all cases. In the original paper, the authors looked at a more complex group of models. However, the competitive risk models they considered can be seen as an extension of the mixture cure rate models presented in this work, specifically when the goal is to model recidivism rates for a particular type of crime. In this context, the authors established three categories: sexual crimes (that is, subjects recidivate for the same cause), violent non-sexual crimes and non-violent crimes. After an exploratory study, the authors considered exponential distribution for the recidivism times of the recidivists from each of the aforementioned categories. In the present work, we have assumed that the exponential distribution is a particular case of the Weibull distribution, and through the estimation of this parameter, we have concluded that for all the cohorts, without separation into categories, the distribution of the recidivism times is not exponential.

Determining if the *r* of the Weibull distribution is equal to, greater or less than 1 essentially means testing the hypothesis that the instantaneous recidivism rates are constant or tend to increase or decrease over time. In fact, if the observed value of the parameter r<1 (as it occurs with our data), then the rates of recidivism at the beginning of the follow-up (1974 in our example) tend to be higher than those observed later (in 1985) among the subjects that until then had not recidivated.

The probabilities shown in Table 4 were obtained by taking the average of the probabilities estimated by the respective models for each individual. For the estimation of probabilities by group, there are at least two different ways to obtain them. The first method consists of taking the averages of the estimated probabilities for each individual in the corresponding group of the sample; for example, the probability of recidivism at three years for those who had NP=0 is the average of the estimated probabilities for the model among individuals that had NP=0. The second method of estimation consists of using all the individuals in the sample and estimating the probabilities that the model would assign them if they had the variables that define the group with the corresponding values and the rest of the variables with their original values. Following the example above, the probabilities for all the individuals are calculated considering their real NPS, AGE and UND16 values, but with NP=0.

In this work, we chose the second method as it gives us the advantage of using data from the entire sample (rather than from a subset) to obtain the estimators. However, using this approach, one must be careful because the value of one (or several) variable(s) can impact the values of others and, consequently, lead to biased estimators. The greater the degree of dependence between the two groups of variables (the conditions that define the group and those that do not), the greater the bias. Consequently, this approach should be used carefully and in conjunction with the exploration of the correlations among the variables. Furthermore, comparisons to the other approach should be considered to check and quantify any potential bias.

It is important to note the two points of information that the mixture cure rate model provides: (1) those who were convicted in 1973 for a crime that victimized a person under 16 years of age had a greater probability of recidivism than those convicted of a crime where the victim was 16 and over years of age, and (2) among the recidivist group, the distribution of the probability of recidivism over time is the same (or independent) for those that committed the crime in 1973 against a minor or against a person 16 and over years of age. This information cannot be obtained with logistic regression or Cox regression models. That the logistic regression model cannot respond is obvious because it cannot incorporate the temporal component, while the Cox regression model cannot perform this because when we incorporate the variable of whether the victim was 16 and over or under 16 and we compare the two groups, we see that in both groups there are as many individuals who recidivate as do not, because of which we cannot compare the two groups solely in terms of those who recidivate. This is an advantage of the mixture cure rate models over the other two models.

This study presents the analysis with a simple dataset composed of only four explanatory variables. However, in certain situations, the information to study recidivism can be composed of a large number of predictor variables. It is not always clear which of these variables has predictive value. The variables that influence the probability of recidivism or the time until this occurs, in general, can be grouped as variables pertaining to the individual, variables of the individual’s inner circle, especially of family, variables of his criminal history, variables of behavior while serving his sentence and of benefits received, including social reinsertion programs. Among the individual variables are age, sex, nationality, educational level, professed religion, the stability of employment and problems of substance abuse, such as alcohol and/or drug use. Variables of the family environment include whether the individual comes from a mono- or bi-parental family, the educational level of the parents, if there is a history of intra-family abuse or substance abuse in the immediate family, the educational level of the parents, if parents and/or siblings have criminal records, marital status, having a stable marital/partner relationship or not and having children or not. Ref. [47] made a review and conducted their own study on the influence of family structure on criminal activity. Other studies in their review have shown that the influence of family structure is small compared to the influence of variables related to family functioning.

Among the variables of the criminal history is the age at which the first crime was committed, the type of crime committed, if the crime was committed under the influence of alcohol and/or drugs and prison sentences received. Among the variables related to behavior during the sentence are the number of acts of misconduct registered and the type of misconduct. Other indicators have also been reported that can be of interest, whether obtained in a psychiatric and/or psychological evaluation or from an evaluation of conduct by a prisoner review board or indices such as the level of criminal commitment. Finally, the variable to be considered can include programs of benefits and/or rehabilitation, the inclusion of which in the models allows for evaluating the effects of such programs on the two basic aspects of recidivism, the probability of recidivating and the average time until recidivating.

Given that the number of models grows exponentially with the number of variables and that it is computationally intractable to fit and test all of them, it is necessary to incorporate variable selection methods in these models. Variables for parametric/semi-parametric cure rate models can be selected through LASSO, Adaptive LASSO, SCAD penalties and related techniques [48,49,50,51,52,53,54,55]. These works considered the expectation-maximization (EM) or the least-angle regression (LARS) algorithms as parameter estimation techniques. However, to our knowledge, these techniques have not been developed for the mixture cure rate models discussed in this article using a Bayesian point of view.

## Figures and Tables

**Table 1:** DIC for the four fitted models.

Model	Without Covariates	With Covariates
Standard Weibull	30,662.57	29,890.05
Weibull–SPM${}_{1}$	30,100.01	29,424.12
Weibull–SPM${}_{2}$		29,780.55
Weibull–SPM${}_{3}$		29,338.96

**Table 2:** Estimates from the Weibull mixture cure rate model (Weibull–SPM${}_{3}$).

	Risk Factor	β	95% Credible Interval (CI)	
Probability	Intercept	0.573	0.357	0.794
of Recidivism	NP	0.532	0.459	0.611
	NPS	0.167	−0.064	0.416
	AGE	−0.405	−0.466	−0.344
	UND16	0.314	0.135	0.493
Survival model	Intercept	0.665	−1.079	2.434
	NP	1.097	0.867	1.318
	NPS	−0.786	−1.842	0.147
	AGE	−1.614	−2.251	−1.015
	UND16	0.061	−1.189	1.345

**Table 3:** Estimate odds ratio (OR) and hazard ratio (HR) by Weibull mixture cure rate model (Weibull–SPM${}_{3}$).

Risk Factor	Long-Term Survival OR (95% CI)	Short-Term Survival HR (95% CI)
NP	1.70 (1.58, 1.84)	3.00 (2.38, 3.74)
NPS	1.18 (0.94, 1.52)	0.46 (0.16, 1.16)
AGE	0.67 (0.63, 0.71)	0.20 (0.11, 0.36)
Age Victim		
≥16	1	1
<16	1.38 (1.15, 1.65)	1.07 (0.31, 3.85)

**Table 4:** Percentages of global and by-group recidivism for the logistic regression, Cox and mixture cure rate models at 3 and 10 years.

	Real	Logistic	Cox	Mixture Cure Rate Model
		Regression	Regression	(Weibull)
Recidivism general	51.9	52.0 (22.1)	47.8 (20.2)	52.6 (21.8)
Recidivism at 10 years	47.5	47.3 (21.8)	47.7 (19.7)	48.1 (22.5)
Recidivism at 3 years	33.4	33.3 (18.5)	33.3 (17.1)	31.7 (18.3)

## Data Availability

The data presented in this study are available from the corresponding author on reasonable request.
